# The Action of Sulphuric Ether on an Enlarged Spleen

**Published:** 1846-02

**Authors:** 


*The action of Sulphuric Ether on an Enlarged Spleen.*—Dr. Corrigan records, in the Dublin Hospital Gazette, a case of ague, in which quinine had been administered with the effect only of prolonging the intervals between the fits. Having given the patient a two drachm dose of ether, combined with twenty drops of laudanum, during the cold stage, the fits did not return; and the following observations of its influence on the spleen were made:

"The margin of the spleen was traced out several times before the measurement was finally taken, or the line of boundry of the organ marked on the surface. This being at length done, it was then found that the space of enlargement of the spleen during the rigor measured six inches in length, and seven inches and a half in breadth. The rigor being still on him he was given two drachms of ether and twenty drops of laudanum. Five minutes after, the rigor had quite disappeared, and the pulse had passed from 120 to 96; skin became warm; and the spleen is now only six inches and three-quarters by four and a half."

Hence Dr. Corrigan concludes—

"1. That the spleen can suddenly alter its volume.

"2. That other agents as well as quinine can effect this sudden alteration of size.

"3. A confirmation of old observation that a cure of the disease can be effected by other remedies as well as quinine."—*Ibid.*

